# Supplementary material for: Are carbon emissions trading and green financial instruments synergistic? -Comprehensive quantitative research based on content analysis
Source: PLoS One. 2024 Mar 7;19(3):e0298601. doi: 10.1371/journal.pone.0298601 (PMC10919601; doi:10.1371/journal.pone.0298601)
Supplement: S1 Data — (ZIP) [file pone.0298601.s001.zip › Data/地级市Z_F工作报告.zip!/╣π╢1⁄2/╒┐╜¡╩╨/└┤╫╘╣1⁄2╓┌║┼í╛┬φ┐╦╩2╛▌═°í┐_2005.docx]

各位代表：

　　我代表市人民政府向大会作政府工作报告，请予审议，并请市政协委员和其他列席人员提出意见。

　　2004年工作回顾

　　2004年是我市全面落实科学发展观，改革开放和现代化建设取得新的重要进展的一年。一年来，在中共湛江市委的正确领导下，在市人大及其常委会和市政协的监督支持下，我们高举邓小平理论和“三个代表”重要思想伟大旗帜，深入贯彻党的十六大、十六届三中、四中全会和胡锦涛总书记视察广东重要讲话精神，以建设现代化新兴港口工业城市为目标，深入实施“工业立市、以港兴市”发展战略，经过全市人民的共同努力，国民经济保持协调健康发展，社会各项事业全面进步，精神文明和政治文明建设取得新成绩，实现了市十一届人大二次会议确定的目标任务，在科学发展道路上迈出了坚实步伐。

　　综合经济实力进一步增强。全市完成生产总值608.16亿元，增长11%，增速创近九年来新高。其中，第一产业增长3.8%，第二产业增长15.4%，第三产业增长10%。人均生产总值9708元，增长10%。

　　工业保持快速增长势头。“工业立市”发展战略有效实施，工业化步伐明显加快。工业实现增加值229.77亿元，增长14%。新增规模以上工业企业75家。规模以上企业实现总产值486.52亿元，增长14.4%；县(市、区)规模以上工业完成总产值243.56亿元，增长20.8%。三次产业比例由上年的21.3∶42.6∶36.1变为20.5∶44.4∶35.1，第二产业的比重提高1.8个百分点。全社会用电量增长20%，其中工业用电量增长22%。实施名牌带动战略取得新突破，红牌、浩特牌电饭煲获“中国名牌产品”，15个产品获“广东省名牌产品”，6个产品获“国家免检产品”，12个商标获“广东省著名商标”，12家企业获美国、欧盟等食品卫生注册认证，10家医药企业通过GMP认证。吴川市被命名为“中国羽绒之乡”。工业增长的质量效益明显提升，工业经济效益综合指数达到223.8%，上升14.5个百分点，居全省第一位；工业对全市生产总值增长的贡献率提高到47.6%，成为经济增长的首要动力。南方大港的优势逐步发挥，全市港口货物吞吐量5096万吨，增长27.9%；其中，湛江港集团完成3124万吨，增长32.4%。

　　农业和农村经济稳步发展。农业总产值206.47亿元，增长4.4%。粮食生产在40年一遇大旱情况下稳中有升，产量154.04万吨，增长3.5%。农业结构进一步优化，农业产业化水平进一步提高。桉树、糖蔗、对虾、珍珠、果菜、畜牧等六大特色产业链不断延伸；43家市级以上农业龙头企业销售收入64亿元，带动农户20.9万户，户均增收2800多元；农产品流通企业发展到1500多家，流通营销额78亿元；农产品加工企业发展到1938家，年加工产值192亿元，其中超亿元企业31家，占46%。农业标准化建设成果喜人，新增两个国家和省级农业标准化示范区；农产品有19个获得国家绿色食品认证，8个评为省无公害产品，39个评为国家和省名优产品，居全省前列；24家水产养殖企业通过省无公害产地认定；廉江红橙获“国家原产地域保护产品”。农业信息化工作取得新成效，“湛江农业信息港”被评为全国百强农业网站。渔业完成产值93.5亿元，其中水产品产值58.5亿元，增长9.7%；积极组织反倾销应诉，湛江国联在全球输美冻虾反倾销案中应诉获胜；北部湾渔民转产转业工作取得新进展，安置渔民1500多人。农村生产生活条件有新改善，实施水利防灾减灾工程14宗，完成投资3.1亿元；完成农村饮水解困工程，基本解决60多万农民群众饮水难问题；建设水库移民新村56条，改造水库移民危房3104户、茅草房3135户。农村税费改革取得明显成效，全市农民人均负担由152.28元下降到8.33元，减负率达94.5%。重大动植物疫病的监控和防治工作扎实推进，抗击禽流感取得阶段性胜利。畜牧业完成产值41.17亿元，增长3.8%。国家级红树林自然保护区建设进展顺利；社会投资造林积极性高涨，全市造林26万亩，其中个体承包造林16.3万亩。

　　投资和消费需求协调发展。全社会固定资产投资150.14亿元，增长31.6%，增幅为近十年来最高水平，发展后劲明显增强。在建亿元以上重点项目22个，实际完成投资48.15亿元，增长56.3%。广东冠豪特种涂布纸、喜利得八厂扩建、中油奥里乳化油库、云广物流中心一期等5个项目建成投产；东兴500万吨炼油改扩建、珠三角成品油管道、包装材料尼龙薄膜、可口可乐和百事可乐湛江分厂等项目即将竣工投产；奥里油发电厂、大中纺织凤梨纤维、德利化油器扩建、徐闻大水桥水库加固等项目进展顺利。全市社会消费品零售总额231.66亿元，增长14.2%。铁路货运量2348万吨，增长36.7%。全年接待游客501.18万人次，增长14.1%；旅游业总收益29.23亿元，增长4.2%。汽车、住房、文化和信息服务等形成消费热点。

　　各项体制改革深入推进。全面实施《湛江市体制改革要点》，41项改革内容完成16项，24项取得实质性进展。市属国有企业产权制度改革加快，17家企业实施资产重组，减少债务3亿多元，资产结构得到优化；15家地方保留糖厂产权改革全部完成，湛江糖业可望走上振兴之路；

　　29家劣势企业退出市场。颁发和贯彻《国有企业领导人员经营决策行为十条禁令》。国有企业分离办社会职能取得成效，湛江港务局实现政企分开，组建湛江港集团有限公司和湛江市港务管理局，港口移交属地管理。城市公用事业改革稳步推进，民营企业有序进入，参与竞争。继续深化行政审批制度改革，清理和取消一批行政许可事项。出台《行政事业性收费项目标准》，规范行政收费行为。颁布《关于促进民营经济发展的实施办法》，有力促进了民营经济发展，新增私营企业1117家，增长9.7%；新增个体工商户1.2万户，增长16.5%；民营经济增加值218.6亿元，增长14.3%。市、县两级政府机构改革全面完成。市直行政单位实行国库集中支付，实现市直机关公务员队伍岗位津贴统发。

　　开放型经济持续增长。实际吸收外商直接投资7136万美元，增长128.3%；合同利用外资2.14亿美元，增长36.5%；规模以上外商及港澳台企业实现工业增加值113.79亿元，增长11.6%；外资大企业、大项目进入增多，已有8家世界500强企业来我市投资或筹办项目。对外贸易保持增长，外贸出口总额9.78亿美元，增长8.6%。其中，高新技术企业出口2亿美元，增长1倍；水产品出口2.7亿美元，增长21.1%。区域合作取得新成效，先后参加泛珠三角区域经贸合作洽谈会、粤港—欧洲经贸合作交流会和首届中国—东盟博览会等，组织召开北部湾经合组织专项交流合作会议。成功举办’2004湛江工业博览会，签订投资合同79宗，合同金额129.9亿元。与澳大利亚凯恩斯市结为友好城市。

　　基础设施和环境建设全面提速。完成市域城镇体系规划编制和城市总体规划修编。积极实施土地储备，处理闲置土地22宗1827亩，累计入库土地6100多亩。湛江港20万吨级铁矿石码头主体工程基本完成，25万吨级航道工程正式开工。渝湛高速公路湛江段、海湾大桥、疏港公路、国道207线良垌至遂溪一级公路等项目建设进展顺利。完成县通镇公路100多公里，镇通村委会公路路面硬化1000多公里。开工建设云头桥公路货运站和雷州雷城、廉江城南汽车客运站，改建危桥30座。市区机场路、椹川大道改造竣工通车；建成金沙湾观海长廊首期工程、东新路和海洋路；完成北桥公园、桥兴园、金康榕园等园林绿化工程，新增绿化面积51万平方米；湖光岩风景区被批准为国家地质公园，并与德国玛珥湖缔结为姐妹湖；完成赤坎污水处理厂首期和冯村垃圾处理场一期工程；霞山污水处理厂开工建设。开展整治违法排污专项行动，市区环境综合质量在全国环保重点城市中排名第3位，环保责任制考核首次跨入省先进行列。

　　财政金融稳健运行。全市财政总收入51.59亿元，增长18.5%；地方财政一般预算收入19.76亿元，增长17.5%；地方财政一般预算支出48.46亿元，增长9.9%，实现财政收支平衡。全市金融机构存款余额605.23亿元，增长12.1%；贷款余额292.5亿元，增长9.7%。

　　各项事业协调发展。科技：新认定省级高新技术企业25家、省级民营科技企业26家。正在实施国家“火炬计划”46项、省“火炬计划”85项、市科技招标项目171项。建立省、市级工程中心16家，市级企业技术开发机构发展到45家，开发新产品208个，在研项目184个；高新技术产品200个，产值87.5亿元，增长40%。与中山大学全面加强校市合作，首批10个项目实施情况良好。教育：市财政投入3500多万元用于市区中小学改造扩容，近两年共有13所学校启动改造，其中6所改造完成，增加学位2400多个；基本完成中小学布局调整，157所老区学校改造全面动工。在省的支持下，基本解决6300多名教师工资欠发问题。文化：遂溪县被命名为“中国醒狮之乡”，遂溪文车高桩醒狮入选上海“大世界吉尼斯纪录”，吴川梅镇被评为全国“民间艺术之乡”，“雷州石狗”列入第一批国家民族民间文化保护工程，雷州市东林村、潮溪村被评为全国历史文化名村。市区开心广场被评为省“十佳文化广场”。成功承办省戏剧演艺大赛和举办市第六届艺术节。市广播电视中心建设进展顺利。第一部《湛江市志》正式出版发行。体育：湛江籍运动员劳丽诗在雅典奥运会勇夺女子双人10米跳台冠军和女子单人10米跳台亚军，实现历史性突破，极大地振奋了湛江人的精神和斗志。成功承办世界第三区(亚澳)滑水锦标赛暨亚洲滑水锦标赛和国家乒乓球队出征雅典奥运会热身赛。成功举办市第十届运动会。卫生：全市参加农村合作医疗人口214.9万人，覆盖率41.7%。实施改造乡镇薄弱卫生院26间。农村卫生户厕普及率达48%，25条村被评为省、市卫生村。建成市、县两级疫情信息网络。人口与计划生育：圆满完成省下达的各项计划生育目标任务，全年人口自然增长率8.32‰；开展“计生优惠政策落实年”活动，农村计划生育家庭奖励部分落实到户。此外，残疾人事业有新发展，市残疾人康复中心建成投入使用。统计、城调、企调部门为经济社会发展提供大量有价值的信息资料，统计信息实现国家、省、市、县四级联网。物价部门加强价格调控、监督和管理，价格秩序不断规范。档案部门加强数字化档案建设，开展现行文件公开阅览服务。外事、侨务、对台等部门扩大与港澳台和国外的经济文化交流。海关、检验检疫部门为我市开放型经济发展作出新贡献。海事部门设立南海海域搜救第一飞行大队，增强粤西海洋搜救能力。气象、地震、减灾防灾、新闻、出版、民族、宗教等各项工作取得新的成绩。

　　人民生活水平有新提高。城镇居民家庭人均可支配收入8921元,增长8.3%;农民人均纯收入3964元，增长7.5%，接近全省平均水平。全市参加社会保险53.4万人，纳入低保3.6万户12.2万人，分别增长2.5%和9%；全年征收社会保险费9.54亿元，增长20.8%；企业离退休人员养老金和失业人员失业保险金全部实现社会化发放；社会保险实现市级统筹。全年新增就业岗位4.85万个，国企下岗失业人员实现再就业2.67万人，其中“4050人员”实现再就业7051人，超额完成全年计划任务。建成村级敬老院120个，解决2035名五保户住房困难。市区建成廉租房2800平方米，部分住房困难群众居住条件得到改善。清理并兑现建设领域拖欠工程款1.7亿元、农民工工资5642万元、征地补偿款4308万元，维护了普通百姓的合法权益。

　　精神文明和民主法制建设取得新成果。开展“新时期湛江人精神”大讨论，培育全市人民积极向上的精神风貌。扎实推进群众性精神文明创建活动，评选命名40条生态文明村，顺利铺开40条特色文化村试点。双拥共建与优抚安置、国防动员、人民防空、兵员征集和民兵预备役工作不断加强，积极为驻地部队解决实际问题，军政军民更加团结，第三次荣获省“双拥模范城”称号。重要决策和重大问题通过人大、政协等渠道征求各界意见，全年办复市人大代表议案及建议222件、市政协委员提案189件。继续开展“振兴湛江——市民创意奖”活动，邀请市民代表参与讨论市政府工作报告。认真贯彻国务院《全面推进依法行政实施纲要》，积极推进依法行政工作。市政府行政服务中心荣获“广东省人民满意公务员集体”称号。组建城市管理行政执法局，相对集中行政处罚权，规范行政执法行为。廉政建设进一步加强，市政府向社会作出并认真履行四项廉政承诺。审计监察加大财政资金、教育资金、国有资产的监督力度，严肃查处一批违纪违法案件。市场经济秩序进一步规范，专项治理教育乱收费、医药购销和医疗服务中的不正之风，强化食品、药品安全监管，切实维护人民群众的切身利益。加强社会治安综合治理，深入开展打恶除霸斗争，依法严厉打击“双抢、两盗”等严重犯罪活动，社会治安保持稳定。保持打击走私高压态势，打私工作受到省的表彰。严密防范敌对势力的渗透破坏，坚决打击“法轮功”等邪教组织的违法犯罪行为。认真处理人民群众来信来访，不断完善人民调解组织和工作制度，多方筹集资金，妥善解决历史积累问题和群众生产生活困难。积极预防并妥善处置群体性、突发性事件，各种社会矛盾逐步化解。安全生产形势稳定，全面完成省下达的控制指标任务。

　　各位代表!过去一年的成就来之不易。这些成就是中共湛江市委正确领导的结果，是各级人民代表大会及其常务委员会和人民政协监督支持的结果，是中央、省驻湛单位和社会各方面关心支持的结果，是全市人民同心同德、艰苦奋斗的结果。在此，我代表市人民政府，向各级人大代表和政协委员，向在各个工作岗位上辛勤劳动、作出贡献的广大工人、农民、知识分子、干部、驻湛人民解放军、武警部队官兵、公安干警和社会各界人士，向所有关心和帮助湛江建设发展的港澳台同胞、海外侨胞和国际友人，向信任和支持政府工作的全市人民致以崇高敬意和诚挚感谢!

　　在充分肯定成绩、共同增强信心的同时，我们清醒地看到，我市经济社会发展与科学发展的要求、与全省经济社会发展的平均水平、与人民群众的愿望还有较大差距，还存在不少困难和问题。一是抢抓发展机遇的意识不够强，经济增长速度达不到全省平均水平，人均生产总值只有全省的49.3%，支撑经济长远发展的支柱产业仍在形成之中；二是农业增效、农民增收的基础仍不牢固，县域经济发展相对滞后，财政乏力，后劲不足；三是外向型经济规模不大，实际利用外资总量偏小，外贸出口面临新的冲击和困难；四是经济社会发展仍有不协调的环节，社会保障能力较弱，就业压力较大，城乡部分群众生产生活比较困难，社会不稳定因素仍然存在；五是城市管理水平仍停留在粗放型阶段，资源保护、环境治理与生态建设任务繁重，投资环境有待进一步优化；六是政府机关作风、办事效率和廉政建设尚未完全适应加快发展的形势，有些关系群众利益的问题还没有得到解决，等等。上述问题和困难告诫我们，实现建设现代化新兴港口工业城市的目标，赶上全省率先基本实现社会主义现代化的步伐，任重而道远。我们必须进一步增强忧患意识、责任意识和竞争意识，戒骄戒躁，艰苦创业，知难而进，高度重视并认真解决存在的问题和困难，迎接新的挑战，开创新的局面，决不辜负全市人民的重托和期望。

2005年工作总体要求和目标任务

　　2005年是我市深入落实科学发展观，保持经济增长、社会进步良好态势，全面实现“十五”计划任务，制定“十一五”规划目标，承前启后的关键一年。做好今年的工作，为新一轮的发展奠定坚实基础，有着十分重要的意义。

　　2005年市政府工作的总体要求是：以邓小平理论和“三个代表”重要思想为指导，以科学发展观统领经济社会发展全局，围绕建设现代化新兴港口工业城市的目标，深入实施“工业立市、以港兴市”发展战略，加快发展县域经济和民营经济，加速工业化进程；推进改革开放，巩固“三农”基础，统筹各方发展，维护社会稳定；坚持以人为本，提高人民生活水平；坚持依法行政，加强政府自身建设，努力赶上全省率先基本实现社会主义现代化步伐。

　　2005年经济社会发展的主要预期目标是：全市生产总值增长10%，其中第一产业增长3.5%，第二产业增长12.9%（工业增长13%），第三产业增长10%；社会消费品零售总额增长12%；固定资产投资增长18%；外贸出口增长10%；实际吸收外资增长10%；地方财政一般预算收入增长10%；居民消费价格涨幅控制在3.8%左右；城镇登记失业率控制在3.5%以内；人口自然增长率控制在7.2‰以下；城镇居民人均可支配收入增长8%；农民人均纯收入增长5%。为实现今年经济社会发展预期目标，必须做好以下工作：

　　一、深入实施“工业立市”战略，加速工业化进程

　　加快重点项目建设。安排亿元以上重点建设项目32个，总投资378.69亿元，今年计划投资55亿元，其中工业重点项目8个。实行重点项目代理制，积极做好土地、资金、供水、供电等建设条件的协调和落实工作，确保重点项目建设顺利进行。力促已竣工的重点工业项目尽快达产增效，并适时增资扩产，形成新的经济增长点。年内第二产业比重超过45%。加快建设奥里油发电厂、大中纺织凤梨纤维等项目。开工建设冠豪2.5亿平方米特种防伪纸、珠江啤酒集团40万吨啤酒、中星石化50万吨沥青项目。积极协同国内投资方落实合作投资主体，力争70万吨木浆项目尽快动工。继续抓好重大项目前期工作，为经济发展积聚后劲。

　　培育壮大重点产业规模。发展壮大临港重化产业。以深水大港为依托，积极引入有实力的企业集团和重大项目；重点抓好石油、化工、纸浆、电力等产业和企业，尤其要支持中石油、中石化、中海油等大型集团在我市扩大投资；大力开发下游产品及相关配套项目，延伸产业链，壮大产业群，形成产业聚集带，推动产业适度重型化。

　　做优做强资源型加工产业。以热带农海产品资源为依托，引进先进技术、工艺、设备和管理，抓好生产加工标准化建设，完善配套服务体系，促进农海产品加工产业上规模、上效益、上水平。大力发展高新技术产业。坚持以信息化带动工业化，实施企业信息化示范工程，加快运用高新技术改造提升传统产业，重点抓好生物医药、新材料、机电一体化、电子信息和海洋资源开发等产业化项目，培育一批拥有自主知识产权、主业突出、核心竞争力较强的高新技术企业，力争高新技术产业产值和增加值分别增长15%和10%以上。

　　激励扶持重点企业发展。继续实施扶优扶强政策措施，推动资产、技术、人才等生产要素向优势企业集中。重点支持销售收入超亿元、税收超千万元的企业做大做强，成为我市工业化的排头兵。支持和鼓励重点企业的技术改造，抓好20项重点技改项目，提高其自主创新和自我发展能力。鼓励争创国家和省名牌产品、驰名和著名商标，争取获得更多国际认证，培育一批名牌企业。积极推动中小企业与龙头企业协作配套，着力培育一批“小而强、小而精、小而专、小而特”的中小企业，形成比较完整的产业增值链和互促互补的产业体系。坚持重点工业企业动态管理，继续公布表彰全市工业企业50强。

　　推进开发区、试验区和工业园区建设。湛江经济技术开发区要谋求新发展，在结构调整、产业发展、对外开放、体制创新等方面实现新的突破；加快推进延伸区和省级高新技术开发区的申报工作，拓展发展空间，更好地发挥排头兵作用。积极谋划东海岛的全面开发，争取国家和省的相关政策支持，完善基础设施，大力引进投资项目，逐步建成承接产业转移的基地。切实抓好省级试验区和工业园区建设，以招商引资为着力点，引导产业链相关的企业向园区集中，逐步形成产业集群，建设具有规模和特色的园区经济。

　　增强县域经济发展活力。创新县域经济管理体制，按省的部署，下放管理权限，增强县级政府对经济社会事务的调控能力。坚持以工业化为核心，突出本地特色，发挥比较优势，大力发展制糖、水产、建材、服装、羽绒、小家电、水果加工等县域支柱产业，千方百计增加县镇两级税源财源。抢抓当前产业转移和广湛高速公路通车的契机，主动吸引珠三角产业转移，共建产业转移园区，力促更多工业项目落户本地。

　　二、切实加强农村工作，提高农业综合生产能力

　　全面落实各项农村政策。贯彻落实今年中央1号文件精神，大力促进农民增收。深化农村税费改革，全面免征农业税。全面完成农村第二轮土地延包30年工作，进一步落实农村土地政策。加强基本农田保护，确保数量不减、质量不降、用途不变。坚持“多予少取放活”方针，全面落实种粮补贴、最低粮食收购价、农资价格监管等各项支农惠农扶农政策，保障农民的利益。继续抓好涉农收费的专项治理，防止农民负担反弹。

　　加快农业产业化步伐。引导和支持农民以市场为导向调整种养业结构，发展效益农业，建设国家级现代化热带农业示范区。提高粮食生产能力，鼓励种粮大户发展规模生产，建设雷州东西洋田现代化粮食生产示范基地；落实粮食生产工作责任制和指导性计划，完成播种460万亩、总产155万吨的粮食生产任务。立足资源优势，选择具有本地特色和市场前景的品种作为开发重点，培育更多农业名牌产品。继续壮大桉树、糖蔗、对虾、珍珠、果菜、畜牧六大优势产业链。启动国家级冬季绿色菜篮子基地建设。重点扶持一批带动性强、覆盖面广的农业龙头企业，以多种利益联结方式，带动基地和农户发展。用2至3年时间，市级以上农业龙头企业发展到50家，带动全市三分之一的农户明显增收。推进农业标准化建设，做好农产品标识与名牌产品属地登记和申报，支持本地名优农海产品申报商标。发展各类农民专业合作经济组织，提高农民的组织化程度。支持湛江农垦增强对全市农业产业化经营的带动作用。依托国家863计划海水养殖种子工程南方基地和湛江开发区水产加工示范基地，促进水产养殖业提档升级。大力发展优质肉牛、肉鸡、生猪的养殖和节粮型畜牧业，提高规模化、集约化饲养水平，壮大畜牧业经济。完成造林24万亩，其中速生浆材林20万亩。

　　优化农村发展环境。完善政府引导、科学规划、市场运作、产业支撑的城镇化机制。坚持统筹城乡发展方略，全面完成19个中心镇的规划修编，落实有关优惠政策，加快中心镇建设。建立稳定增长的支农资金渠道，逐步增加农业投入，推进城乡水利防灾减灾工程和农田水利、机电排灌、抗旱水源工程建设，提高农业抗御自然灾害的能力。继续抓好农村饮水解困工程。全面完成县通镇二级公路建设任务，建设镇通行政村公路路面硬化700公里，改造危桥30座，建设5个县（市）级枢纽站场。完善农业科技推广服务体系，开展针对性强、务实有效、通俗易懂的农业科技培训，加大良种良法的推广力度。探索建立满足农民和农村需要的小额信贷组织，加快落实对农户和农村中小企业实行多种抵押担保的有关规定。推进海洋功能区划编制工作，加强海岛渔区基础设施建设，启动硇洲国家级中心渔港工程，稳步推进北部湾渔民转产转业。继续实施农村茅草房改造和水库移民安居工程，建设移民新村57条。

　　积极推进农村劳务输出。加强农村劳动力就业的教育和引导，树立吃苦耐劳、艰苦创业精神，切实做好农村劳务输出的组织指导、管理培训、就业信息咨询和服务维权等工作，积极输出农村富余劳动力，拓宽农民增收渠道，年内转移农村劳动力19万人。

　　三、深化各项改革，增强发展动力

　　全面落实《湛江市体制改革要点》。坚持加快推进，综合配套，不断完善，落实各项改革内容；对每项改革工作要明确领导和责任单位，采取有力措施，年内取得明显成效。

　　深化国有、集体企业改革。深入推进产权制度改革，加快投资主体多元化进程，促进各类资本的流动、重组、融合，大力发展国有、集体和非公有资本参股的混合所有制经济。不断完善国有资产监管体系，全面推行年度经营业绩责任制和经营者任期业绩考核责任制。推进国有企业资产重组，扶持10家以上优势国有企业做大做强，鼓励和推动优势企业改制上市融资。加快劣势国有、集体企业退出市场。支持境内外投资者和民营企业参与国有企业改组改造。加快国有粮食企业改革和供销合作社产权制度改革。推进县级供电企业管理体制改革，继续整顿农村电价。

　　加快发展民营经济。全面落实国务院《关于鼓励支持和引导个体私营等非公有制经济发展的若干意见》，贯彻平等准入、公平待遇原则，凡是法律法规未禁入的行业和领域全面向非公有资本开放，并放宽股权比例限制，在投资核准、融资业务、财税政策、土地使用、对外贸易和经济技术合作等方面，与其他所有制企业一视同仁，给予大力支持。积极引导民营企业创立名牌、创新技术、规范管理，壮大经济规模，提升发展水平，培育一批市场竞争力强、辐射带动作用突出的民营大企业、大集团。依法加强监督和管理，切实保护民营企业的合法权益。评选表彰优秀民营企业和优秀民营企业家。

　　创新城市公用事业运营机制。进一步开放市政公用事业建设和运营市场，加快公共交通、园林绿化、环卫、供水、燃气、污水及垃圾处理等行业的市场化步伐。放宽城市公用事业投资准入条件，鼓励和引导民资、外资以各种形式参与建设和经营。选择一批具有较好经济效益的市政项目，向社会投资主体公开转让经营权。理顺公共产品价格，建立既有利于鼓励社会和外商投资，又兼顾到市民承受能力的价格机制。

　　健全和完善公共财政制度。进一步完善国库集中收付制度，严格执行“收支两条线”管理规定，加强对基金、收费征缴各环节的管理，全面开展财政资金使用绩效评价，提高财政资金使用的规范性、有效性和安全性。加快部门预算改革，将行政部门预算定员定额管理推进到市直事业单位。推广财政性资金招标使用办法。强化税收征管。新增财力主要用于关系长远发展的基础性项目建设、保障社会稳定和解决困难群众生产生活问题。进一步清理和处置市直党政机关、事业单位的资产。出台《湛江市区财政管理体制改革实施方案》，合理划分市与区的财权关系，调动市辖区生财理财的积极性。

　　推进金融业改革和发展。大力支持中央和省驻湛金融机构发展壮大。加强银企沟通和协调，推动银企合作，拓展金融信贷市场。按照统一部署，改革农村信用社管理体制和产权制度，强化约束机制，增强服务功能。加大中小金融机构和各类基金会资产清收力度，提高防范地方金融风险能力。

　　四、扩大对外开放，拓宽发展空间

　　保持外贸出口有效增长。转变外贸增长方式，壮大外贸出口规模。注重提高企业出口竞争力，培育和发展多种所有制并存的出口基地；注重拓展东盟各国和中东、拉美、大洋洲等市场；注重优化出口商品结构，大力培育自主品牌产品，提高出口附加值；注重发展加工贸易和进口贸易。推进电子口岸建设，优化通关服务环境。加快建立应对国际贸易壁垒的预警机制，提高抵御国际贸易市场风险的能力。每个县(市、区)年度完成外贸出口2000万美元以上，力争一至两个县(市、区)超1亿美元。

　　进一步扩大利用外资。抓住国际资本和产业加快向我国沿海港口城市转移的重大机遇，加大对外推介力度，吸引更多外资投向我市的重化工业、现代农业和现代服务业。积极跟踪落实签约重点外资项目，重点支持粤海中纤板等项目增资扩产，支持华丽金三期、国联水产三期、信威工艺二期等项目加快建设。吸引更多跨国公司特别是世界500强企业前来投资。充分发挥企业的主体作用，调动各方面招商引资的积极性。创新招商引资方式，大力推行网上招商、委托招商、园区招商等多种形式，提高招商引资工作的实效。落实招商引资责任制，各县（市、区）年度吸收外商直接投资1000万美元以上。

　　推动区域经济合作。充分利用我市作为大西南主要出海通道的有利条件，积极参与泛珠三角区域经济合作，扩大与经济发达地区的经济联合协作，积极与西南各省区市联手进行资源开发、产业对接、市场开拓和发展物流业。加强与东盟、香港、澳门及北部湾周边城市、友好城市的经贸合作，进一步提升区域合作发展层次和水平。办好'2005湛江工业博览会。

　　大力发展商业和现代流通业。认真制定全市商业物流业发展规划。重点抓好沃尔玛购物广场、国贸城市广场、云广物流中心二期等项目建设，支持霞山水产品批发市场等流通骨干企业和专业批发市场发展壮大，推动专业批发市场由商品集散地向物流配送中心转型。筹建本地名优产品展示交易中心。发展连锁经营、电子商务、超级市场等新型流通业。支持湛江港发展港口物流业、航运业，加快物流园区建设，引进国内外大型物流企业，建设矿石、石油、粮食、化肥、糖料、木材等大宗物资中转基地，全年实现港口货物吞吐量6000万吨。积极引导房地产业适度健康有序发展，激活二级市场，促进住房消费稳步增长。

　　加快发展旅游业。大力宣传“相约在中国大陆最南端”的旅游品牌，扩大“中国优秀旅游城市”的效应。以独特的红土文化、蓝色海滨、绿色生态为主线，加强景点景区和星级酒店及配套设施建设，提高服务质量。积极走出去举办旅游推介会，加大引客入湛力度。保护开发火山峡谷群和徐闻近海珊瑚礁资源。湖光岩风景区着手申报世界地质公园。加快雷州国家级历史文化名城建设，保护利用市区历史文化建筑和农村历史文化村，丰富特色旅游的文化内涵，提升旅游品位。

　　五、推进基础设施和环境建设，增强可持续发展能力

　　加快以港口为重点的基础设施建设。齐心协力建设南方大港，支持湛江港集团不断增强自身实力和竞争力。年内完成湛江港20万吨级铁矿石码头和25万吨级航道工程；启动港区粮食码头和铁路改造等项目；做好宝满港区集装箱、散装化肥和东海岛港区码头建设前期工作。进一步推动大港口与大交通的衔接，年内建成疏港公路和渝湛高速公路湛江段、国道207线良垌至遂溪段；加快海湾大桥及西连接线52公里道路建设；力促洛湛铁路茂湛段、黎湛铁路复线湛江段和同三高速公路湛江至海安段早日动工。加快建设500千伏港城输变电工程。

　　推进市政环境建设。加快渔港公园和寸金路续建工程建设，确保今年国庆节前完成。新建南国热带花园、绿塘河生态公园、麻章百乐园、坡头灯塔公园；改造霞湖公园、湖光岩风景区、三岭山森林公园、东坡荔园和赤坎水库生态园；推进滨湖公园前期工作。建设体育南路、站前路、东盛路和金沙湾观海长廊二期工程；继续改造、整治市区小街小巷。完成霞山污水处理厂一期和冯村垃圾处理场二期工程，建设赤坎污水处理厂二期工程，筹建麻章污水处理厂。继续规划建设市区垃圾转运站和卫生公厕。量力而行推进旧城区和城中村改造。动工建设市区45公里堤防工程，配套建设道路、景观、休闲等设施。

　　加强生态建设和环境保护。推进林业生态县建设，加快营造霞山、赤坎观海长廊红树林带；大力开展创建绿色住区及实验小区活动，市区年内增加绿化面积50万平方米。继续创建国家园林城市、国家卫生城市和国家环保模范城市，争取2-3年内实现创建目标。 编制生态保护建设规划，合理适度开发水资源，抓好九洲江水系和鉴江、南渡河水资源的合理开发和保护。加快海洋与渔业自然保护区建设。加强生态农业建设和农村环境保护。实施治污保洁、蓝天碧海和宁静工程，启动清洁生产示范试点，整治违法排污，推动工业污染源全面达标。加大固体废弃物、医疗废弃物、放射性和噪声等污染防治。继续治理城市卫生死角和国道、省道两旁的卫生环境，加强机动车尾气管理，加快饮食服务业油改气进程，开工建设湛江电厂脱硫工程，改善城市大气质量。

　　提升城市管理水平。建立完善市、区、街与部门之间相互衔接、合理分工、规范高效的城市管理体制。进一步推动城市管理重心下移和职责到位，全面实行城管责任监督。依法加强规划监督管理，实行违规工程项目定期公告制度，向社会公开违法违规建设工程项目清理情况和处理结果，维护城市规划的权威性。加大城市管理综合执法力度，严厉整治交通秩序和市容卫生等突出问题，进一步改变市容市貌。

　　六、按照建设文化大市要求，统筹发展社会各项事业

　　促进教育事业发展。继续完善以县为主的义务教育管理体制，巩固“普九”成果，保障教职工工资待遇。坚决治理教育乱收费，采取有效措施解决群众上学难、上学贵问题。加快改造校舍危房，年内完成157所老区学校改造。推进市区学校扩容增班，继续解决大班额问题。加快湛江一中初中部和市二中新校区建设，创办示范性高中，提高高中入学率。扶持社会力量办学。切实解决英语、信息教育师资严重缺乏问题，全面提高基础教育质量。加快基础教育信息化进程。筹建工读学校。

　　促进科技事业发展。申报组建省级高新技术产业开发区，推进国家火炬计划湛江海洋产业基地、省级农业科技园、省级可持续发展试验区、生态环境示范基地和专业镇试点建设。继续实行科技计划项目公开招标。开展重点工业企业知识产权试点工作，完善以企业为主体的科技创新体系，增强企业自主创新能力，争取全年专利申请量和授权量增长15%以上。扩大与高等院校的科技合作，积极利用高校科研成果为我市经济发展服务。鼓励我市高校师生开展科技试验、科技发明、科技创业活动。

　　促进文化事业和文化产业发展。建设省重点扶持艺术创作基地，发展地方特色戏剧，积极参与东西两翼基层文化建设扶持工程，筹建市文化艺术中心。打响湛江传统文化和民间艺术品牌，举办湛江红土艺术节和国际醒狮邀请赛，推介人龙舞、飘色等民间艺术，在广大农村倡导先进文化节活动。深化文化体制改革，扶持发展新闻和文化产业，鼓励民营资本参与兴办国家政策许可的文化项目，培育一批重点民营文化企业。强化文化市场综合执法，整治中小学周边环境，加强网吧、游戏机室等文化经营场所及媒体广告的监管。增建青少年课外文娱活动场所，完成市广播电视中心建设，加快有线电视数字化进程，继续做好自然村“村村通广播电视”工作。

　　促进卫生体育事业发展。深化城镇医疗体制改革，引导卫生资源向社区、农村流动和配置，大力发展基层卫生服务。倡导和推广平价医院、平价诊室和平价药房，为低收入群众提供廉价实惠的医疗服务，努力解决群众看病难、看病贵问题。加强120急救中心网络建设，筹建市传染病医院。做好非典、禽流感、艾滋病等重大传染病的预防控制工作。进一步完善无偿献血网络，确保临床用血的需要和安全。推进乡镇薄弱卫生院改造。加快建立县级统筹农村合作医疗保障制度，年内实现农村人口参加合作医疗覆盖率50%以上。健全妇幼保健体系，提高妇女儿童保健水平。积极备战2006年省第十二届运动会，推动全民健身运动的广泛开展。挖掘场馆潜力，开发体育产业。争创体育先进县（市）、先进社区和先进镇。加强中小学体育工作。

　　促进人才资源开发。实施人才强市战略，加强专业技术人才队伍建设。积极引进本科学历以上毕业生和留学回国及海外人才，努力开发本地人才资源。做好大中专毕业生就业服务工作。推动人才流动服务机构体制改革，完善人才市场体系，消除人才流动的体制性障碍，促进形成全市性人才市场。

　　促进人口与计生工作。全面落实计划生育政策，提高出生人口素质。认真落实计划生育目标管理责任制，坚持“一票否决”和重心下移的工作机制。对农村计划生育家庭实行奖励扶助。加强计划生育服务网络建设。切实抓好人口抽样调查工作。综合治理出生人口性别比失衡问题。加快人口计生系统信息化建设。巩固殡葬改革成效，坚决制止殡改中的不正之风。

　　全面做好各项社会工作。积极推进全民国防教育，增强全民国防意识，做好国防动员和民兵预备役、人民防空工作。深入开展双拥共建活动，做好优抚安置，热情帮助驻湛部队解决实际问题。完善统计巡查制度，认真开展第一次经济普查。推行价格听证，健全价格监测预警机制。强化审计监督，加大财政资金和国有资产的监管力度。推进省人工影响天气湛江基地建设。推动第二届地方志修编。筹建市级综合档案馆，推进数字化档案建设。进一步做好民族、宗教、外事、侨务、对台、老龄等工作，促进社会各项事业协调发展。

　　七、努力纾解民困，促进社会公平

　　深入实施“十项民心工程”。继续认真实施全民安居、扩大与促进就业、农民减负增收、教育扶贫、济困助残、外来民工合法权益保护、全民安康、治污保洁、农村饮水和城乡防灾减灾十项民心工程，务必取得实实在在的成效，使经济发展和社会进步的成果惠及全体百姓。

　　千方百计扩大就业和再就业。完善“劳动者自主就业、市场调节就业、政府促进就业”的就业机制，强化就业再就业目标责任制考核。落实小额担保贷款、社保和岗位补贴专项资金等再就业扶持政策。动员社会各方力量，积极开辟就业门路，全年新增城镇就业岗位3万个。广泛开展困难群众就业援助活动，力争城镇“零就业家庭”半年内有1人就业。建立健全覆盖城乡的劳动力市场信息网络，规范劳务收费。发展技工教育，加强职业技能培训。重点做好转制国有企业职工再就业、复员转业军人安置、高校毕业生和城镇新增劳动力就业工作。

　　健全社会保障体系。继续扩大社会保险覆盖面，提高非公有制企业社会保险参保率。继续推行社会保险市级统筹，完善医疗、失业、工伤保险制度，启动女职工生育保险。强化基金征缴和监管，推行地税部门全责征收社保费。全面完成乡镇（街道）劳动保障工作机构建设任务。健全城乡最低生活保障制度，年内新增享受低保的城乡居民3万人，力争应保尽保。鼓励发展公办民营、民办公助等多种形式的福利事业。

　　做好扶贫济困助残工作。认真实施扶贫规划，大力开展机关、事业单位定点扶贫和社会力量扶贫，帮助农村贫困户实现人均年收入2000元以上，力争40%以上有青壮劳动力的农村贫困户实现1人转移就业。切实管好用好各项扶贫资金。建立健全城乡低收入困难群众社会救助体系，着力帮助他们解决实际困难。做好征地拆迁中的补偿安置工作，继续清理拖欠征地补偿款和建设领域拖欠工程款、农民工工资，防止出现新的拖欠。妥善安排农村“五保户”和特困户的生活，年内增建150间村级敬老院。城区继续建设廉租房。全面实施“明天计划”和“扶残助学”工程，为残疾人倾注关爱，促进残疾人事业发展。积极解决优抚对象、困难企业军转干部、伤残军人以及特殊困难群众生活保障问题。

　　八、加强精神文明和民主法制建设，构建和谐湛江

　　扎实推进精神文明建设。深入开展“爱国、守法、诚信、知礼”现代公民教育活动，引导全社会形成热爱祖国、遵纪守法、诚实守信、知书达礼的道德风尚和行为规范。切实加强未成年人思想道德建设。积极创建全国文明城市先进单位和文明县(市)城区，力争有1至2个县(市)城区进入省文明城区行列。广泛开展创建文明行业、文明单位活动，重点抓好窗口行业、执法部门和“诚信企业”的创建工作。推动生态文明镇建设，重点抓好徐闻县曲界镇、雷州市英利镇、廉江市良垌镇三个示范点。深入开展文明社区、生态文明村和特色文化村创建活动。

　　加强社会主义民主建设。自觉接受人大及其常委会的监督，支持人民政协履行职能，认真办理人大代表议案、建议和政协委员提案，主动加强与各民主党派、工商联、人民团体、无党派人士的联系，充分发挥工会、共青团、妇联等组织的桥梁纽带作用。完善以职工代表大会为主要形式的企业民主管理制度。普遍推行村务、厂务和政务公开，扩大公民有序的政治参与，进一步扩大基层民主。上半年完成村(居)委会换届选举。抓好基层“民主法治示范村”创建工作。加强法制宣传教育，扩大法律援助，做好行政复议、行政诉讼工作，提高政府和公民运用法律解决问题的能力。通过法定渠道加强对政府机关和工作人员的监督。

　　高度重视信访工作。认真贯彻执行国务院《信访条例》，完善信访工作领导责任制以及联席会议、排查调处和督查工作制度。进一步落实领导接访、下访、约访群众制度，畅通信访渠道，积极化解和妥善处理人民内部矛盾、特别是涉及群众切身利益的矛盾。依法规范群众上访行为，建立良好信访秩序。加强督查督办，提高信访案件结案率。充分发挥人民调解、行政调解和司法调解的综合作用，及时发现和化解各种苗头性、倾向性问题，努力把问题解决在萌芽状态，解决在基层。继续办好市长专线电话和电子邮箱，及时倾听市民意见。

　　规范市场经济秩序。建立和完善市场经济秩序综合治理长效机制。规范市场价格行为，加强对重点行业、特种行业的监管。全面推行食品、药品市场安全准入制度，加强农产品质量安全监测，实施“食品放心”工程，严格监管食品市场。规范运输市场秩序，为乘客和货主提供安全优质服务。推进诚信社会建设，促进企业守法经营。加大打假治劣力度，依法严厉打击制售假冒伪劣商品行为。全面治理“三乱”行为，年内实现“公路无三乱城市”目标。规范各类市场中介组织行为。

　　抓好社会治安综合治理。坚持打防结合、预防为主，建立完善社会治安综合治理长效机制。严厉打击各类违法犯罪活动，特别是黑恶势力犯罪、严重暴力犯罪和贩毒、走私犯罪以及“双抢、两盗”等多发性侵财犯罪。加强禁赌工作，重点打击“六合彩”、“私彩”等违法犯罪行为，严肃查处领导干部、国家公务员、企事业单位负责人参与赌博或赴境外参赌问题。开展社会治安重点地区、重点部位专项整治行动。加强国家安全工作，取缔各类邪教组织，严密防范和有效打击各种敌对势力的渗透破坏活动。抓好劳教工作，重视做好刑释解教人员的安置和帮教。

　　切实加强安全生产。全面落实安全生产责任制，建立和完善防范重特大安全生产事故的责任与追究机制。加大安全生产执法力度，排查整改安全事故隐患，重点开展道路与水上交通、危险化学品、人员密集场所、烟花爆竹与民爆器材等安全专项整治，消除重大安全隐患。抓好安全标准化示范工作，增加公共安全设施投入。全面落实省下达的安全生产控制指标，遏制重特大事故发生，稳定安全生产形势，确保人民生命财产安全。

　　九、编制“十一五”规划，谋划新的发展

　　各级各部门对“十一五”规划编制工作要高度重视，精心组织。坚持以科学发展观为指导，把以人为本、全面协调可持续发展的理念贯彻规划始终，体现进一步落实全面建设小康社会、建设现代化新兴港口工业城市和打造“一个中心、五个基地”(粤西地区和环北部湾经济圈的区域性中心城市，重化能源基地、区域性物流基地、海产品生产加工基地、热带农业示范基地和特色文化旅游基地)的目标任务。认真调查研究，深入分析论证，广泛征求意见，充分集中民智，力求使规划具有较高的预见性、科学性和指导性。年底前完成规划纲要编制工作，明年初提交市人民代表大会审议。

切实加强政府自身建设

　　面对新形势、新任务，各级政府必须以党的十六届四中全会精神为指导，按照科学执政、民主执政、依法执政和人民满意的要求，进行“理想、责任、能力、形象”教育，全面提高政府的服务意识、行政能力和管理水平。

　　以提高行政能力为取向，建设法治政府。认真贯彻落实《行政许可法》和《全面推进依法行政实施纲要》，加强政府法制工作，提高政府工作人员特别是领导干部依法决策、依法办事能力。强化行政执法层级监督，建立行政执法责任制。制订实施《行政效能监察办法》，明确监察内容、监察对象和监察结果的处置原则。实行责任追究制度，将政府系统各级各部门的行政首长列为问责对象，明晰行政机关的权责关系，促使政府机关及其负责人对自身的行为及其后果负责，激励做出正确的政绩、实绩，最大限度地制止失职、失误，做到有权必有责，用权受监督，侵权须赔偿，违法要追究。

　　以转变职能为取向，建设有为政府。适应市场经济体制的要求，进一步创新政府管理的方式方法。致力于创造良好的经济发展环境，维护公正、公平、公开的市场秩序，最大限度地减少行政手段，更多地培育并运用经济和法律的手段管理社会。加快政企、政事、政资分开，坚决把政府不该管、管不了也管不好的事，进一步交给企业和市场。大力维护社会公正和社会稳定，完善社会利益协调机制，逐步解决地区之间和部分社会成员之间收入差距过大问题。

　　以利民便民为取向，建设服务政府。要增强民本意识，常怀爱民之心，恪守为民之责，多谋利民之策，做到民有所呼、必有所应，民有所忧、必有所虑，民有所难、必有所助，民有所求、必有所为，时刻关注民生，努力纾解民困，让全体人民共享社会发展的成果。继续深化行政审批制度改革，办好各级行政服务中心，推行“一站式”、“一窗口”服务方式，简化手续，减少环节，提高工作效率和服务质量。落实层级责任，实行收文办结定期公开和市分管领导督办制，及时解决群众投诉的问题。

　　以扩大市民参与为取向，建设透明政府。把科学决策、依法决策、民主决策作为政府决策的基本准则，增强政府决策和行政的透明度，拓宽社情民意的反映渠道，健全人民群众参与决策的途径和形式。涉及经济社会发展全局的重大事项，广泛听取社会各界的意见；对关系人民群众切身利益的重大问题，注重充分协商和协调，及时向社会公告或组织听证。规范政务公开制度，增加政务公开内容，政府投资项目、国有土地划拨实行公示，学校、医院和城市公用事业等与群众利益密切相关的公共部门和单位，要全面推行办事公开制度。继续开展“振兴湛江—市民创意奖”活动。邀请市民旁听政府全体会议。试办政府新闻发布会，继续办好政务公示栏，向社会免费发放刊载政府规范性文件的《湛江政务》。通过广泛的政务公开，把政务行为置于人民群众的直接监督之下。

　　以增强制度约束为取向，建设廉洁政府。全面落实廉政建设责任制，探索建立健全教育、制度、监督并重的惩治和预防腐败体系。加强对公务员的教育和管理，认真履行政府廉政承诺，严格执行廉洁从政的各项规定。对施政行为的全过程实施有效制约监督，尤其要加强对群众反映强烈的社会热点难点问题和掌管财物等部门的监督，严肃查处违纪违法案件。当前，要特别重视做好财政性资金的管理工作，确保预算的严肃性、资金的安全性和管理的规范性，坚决杜绝和制裁挤占、挪用甚至私分财政资金的违法犯罪行为。严肃对待政府审计工作，对违规问题要认真整改，年内实行审计情况和整改结果向社会公告。

　　各位代表!湛江正在加快发展的航程中奋进。我们对湛江的美好前景充满信心。让我们高举邓小平理论和“三个代表”重要思想的伟大旗帜，牢固树立和认真落实科学发展观，在中共湛江市委的坚强领导下，同心同德，再接再厉，奋发图强，为加快建设现代化新兴港口工业城市和美丽的南方海滨城市，为全市710万人民实现富裕安康而努力奋斗!
